# Effects of Local Acidification on Benthic Communities at Shallow Hydrothermal Vents of the Aeolian Islands (Southern Tyrrhenian, Mediterranean Sea)

**DOI:** 10.3390/biology11020321

**Published:** 2022-02-17

**Authors:** Emanuela Fanelli, Simone Di Giacomo, Cristina Gambi, Silvia Bianchelli, Zaira Da Ros, Michael Tangherlini, Franco Andaloro, Teresa Romeo, Cinzia Corinaldesi, Roberto Danovaro

**Affiliations:** 1 Department of Life and Environmental Sciences, Polytechnic University of Marche, Via Brecce Bianche, 60131 Ancona, Italy; sdgaron94@gmail.com (S.D.G.); c.gambi@univpm.it (C.G.); silvia.bianchelli@univpm.it (S.B.); z.daros@pm.univpm.it (Z.D.R.); r.danovaro@univpm.it (R.D.); 2 Stazione Zoologica di Napoli Anton Dohrn, Villa Comunale, 80100 Naples, Italy; michael.tangherlini@szn.it (M.T.); franco.andaloro@szn.it (F.A.); teresa.romeo@szn.it (T.R.); 3 Department of Materials, Environmental Sciences and Urban Planning (SIMAU), Polytechnic University of Marche, Via Brecce Bianche, 60131 Ancona, Italy; c.corinaldesi@univpm.it

**Keywords:** ocean acidification, shallow hydrothermal vents, meiofauna, macrofauna, biodiversity, Mediterranean Sea

## Abstract

**Simple Summary:**

Ocean acidification is causing major changes in marine ecosystems, with varying levels of impact depending on the region and habitat investigated. Here, we report noticeable changes in both meio- and macrobenthic assemblages at shallow hydrothermal vents located in the Mediterranean Sea. In general, the areas impacted by the vent fluids showed decrease in the abundance of several taxa and a shift in community composition, but with a clear biomass reduction evident only for macrofauna. CO_2_ emissions at shallow hydrothermal vents cause a progressive simplification of community structure and a general biodiversity decline due to the loss of the most sensitive meio- and macrofaunal taxa, which were replaced by the more tolerant groups, such as oligochaetes, or highly mobile species, able to escape from extreme conditions. Our results provide new insight on the tolerance of marine meio- and macrofaunal taxa to the extreme conditions generated by hydrothermal vent emissions in shallow-water ecosystems.

**Abstract:**

The Aeolian Islands (Mediterranean Sea) host a unique hydrothermal system called the “Smoking Land” due to the presence of over 200 volcanic CO_2_-vents, resulting in water acidification phenomena and the creation of an acidified benthic environment. Here, we report the results of a study conducted at three sites located at ca. 16, 40, and 80 m of depth, and characterized by CO_2_ emissions to assess the effects of acidification on meio- and macrobenthic assemblages. Acidification caused significant changes in both meio- and macrofaunal assemblages, with a clear decrease in terms of abundance and a shift in community composition. A noticeable reduction in biomass was observed only for macrofauna. The most sensitive meiofaunal taxa were kinorhynchs and turbellarians that disappeared at the CO_2_ sites, while the abundance of halacarids and ostracods increased, possibly as a result of the larger food availability and the lower predatory pressures by the sensitive meiofaunal and macrofaunal taxa. Sediment acidification also causes the disappearance of more sensitive macrofaunal taxa, such as gastropods, and the increase in tolerant taxa such as oligochaetes. We conclude that the effects of shallow CO_2_-vents result in the progressive simplification of community structure and biodiversity loss due to the disappearance of the most sensitive meio- and macrofaunal taxa.

## 1. Introduction

The atmospheric carbon dioxide (CO_2_) concentration, due to anthropogenic activities, has increased from 280 ppm in preindustrial times to a present-day level of ~418 ppm [1]. This increase also results in dramatic effects within the oceanic realm, by reducing carbonate saturation of estuarine, coastal, and surface open-ocean waters [2] and expanding the volume of acidified seawater [3]. Previous investigations have been conducted at shallow submarine hydrothermal vents around active volcanoes (here referred to as hydrothermal CO_2_ seeps), where large amounts of CO_2_ are injected into seawater [4]. They also showed different impacts of ocean acidification (OA) on marine life, especially on calcifying species such as corals [5], but also on seagrasses, macroalgae [6,7], and meiofauna [8], which are fundamental in the benthic food-web, being responsible for the matter and energy transfer to higher trophic levels [9,10].

Seawater acidification in shallow CO_2_ vents can alter the diversity of calcareous algae and invertebrates near sites at lower pH with signs of dissolution of gastropods shells [10]. In addition, meiofaunal abundance and diversity are negatively influenced by the presence of shallow-water hydrothermal vents as documented in the Southern Pacific Ocean [11]. Moreover, OA leads to a simplification of food webs with the trophic structure of the invertebrate community shifting to fewer trophic groups and the dominance by few generalists in low pH conditions [12].

In the Aeolian Arc (Aeolian Islands, Southern Tyrrhenian sea) in particular, the metal-rich geochemical composition, the presence of chemosynthetic bacteria, and the emission of CO_2_ fluids enriched with toxic chemicals create the so-called “Smoking Land”, i.e., a complex of ca. 200 volcanic vents located between the Panarea island and the islet of Basiluzzo, a highly interesting area and a natural laboratory for studying the effects of acidification on marine ecosystems [13] and in particular on benthic communities.

Benthic fauna provides crucial insights on the health status of marine ecosystems and information on habitat changes and/or alteration due to disturbance events [14,15]. In particular, soft-bottom infauna (either macrofauna and meiofauna), due to its limited mobility and intimate association with seabed, is directly exposed to the changing environmental conditions and is sensitive to most chemical and physical alterations.

Macrobenthic invertebrates have been repeatedly identified as important biological components to assess the environmental status of marine ecosystems due to their ecological role and importance in marine food webs and nutrient cycling [16,17]. For this reason, macrofauna have been the most investigated component for the assessment of the environmental impacts and are considered one of the most effective indicators of changing environmental conditions [18].

Meiofaunal organisms are good indicators of environmental conditions, and changes in their abundance, community structure, and functional diversity may indicate alterations in the system [19,20]. Thanks to their ecological characteristics, meiofauna as bioindicators present some advantages over macrofauna (e.g., ubiquity, high abundance and diversity, small size, short life cycles, limited mobility, absence of pelagic life stages, and the presence of both tolerant and sensitive taxa/species), because they respond rapidly to changes in disturbance in aquatic ecosystems [19,20].

Meiofauna and macrofauna respond differently in time to natural and anthropogenic disturbance, with meiofauna usually showing a quick response to changes in the ecosystem and also a rapid recovery when compared to macrofauna. Meiofauna indeed show high turnover rates, rapid reproduction, and rapid life histories [21]. By integrating both meiofauna and macrofauna in environmental disturbance assessments, either due to natural or anthropogenic events, our understanding of the impacts on the benthic community in particular, as well as marine ecosystems, is greatly improved.

In the present study, we hypothesize that the presence of hydrothermal vents influences sediment properties, and consequently meio- and macrofaunal assemblages in terms of abundance, community composition, and diversity. This study aims to assess: (a) the effect of local acidification on the abundance, biomass, species composition, and assemblage structure of meio- and macro-fauna communities; (b) the presence of highly sensitive taxa, which might provide indication on the long-term effects of OA; and (c) the effect of depth on the benthic community patterns observed.

## 2. Materials and Methods

### 2.1. Study Area

The hydrothermal system of Panarea island is ca. 70 km^2^ and includes active vents characterized by an intense exhaust of gases dominated by CO_2_ and thermal fluids with temperatures up to 140 °C [22,23]. This hydrothermal system suffered a sharp increase in ventilation activity in November 2002 due to the injection of magmatic fluids into the deep geothermal body that caused a low-energy underwater explosion, killing almost all living organisms in the area [24]. The magma uplift in the nearby volcanic island of Stromboli allowed the hot magmatic volatile compounds to migrate to Panarea through the normal active fault that connects the two volcanic structures [25]. Around the islet of Basiluzzo, hydrothermal processes produce sulfide deposits and vents rich in Fe [26]. Despite the numerous and detailed information on the geomorphological characteristics of the Panarea volcanic complex, investigations on the biological components of this underwater hydrothermal system are scant and still ongoing. Recent studies have highlighted the presence of structures with different shapes and sizes with diffuse gas and thermal emission. The presence of many active and different conformation vents has given this area the name “Smoking Land” [26]. The volcanic complex of Panarea represents the emerging part of a composite submarine volcano belonging to the Aeolian arc in the southern Tyrrhenian Sea [27]. Remains of primary volcanic structures can be traced to “Secca dei Pesci”, to the S and SW areas of Panarea and to the NW and NE areas of Basiluzzo [26]. Between the island of Panarea and the islet of Basiluzzo, the presence of gas leakage, white spots, bacterial mats, and precipitates of Fe-oxyhydroxides has been detected [28]. About 200 vents with different shapes and sizes have been characterized by ROV [26]. Among all the volcanic vents observed, some are clearly active, with the emission of bubble plumes or hydrothermal fluids having a density visibly different from the surrounding seawater [26]. The origin of the “Smoking Land” can be attributed to the presence of a strong hydrothermal circulation along the fault plane of the north-western graben. Acid fluids enriched in dissolved inorganic carbon (DIC) and trace elements for volcanic degassing emerge from the bottom and are accompanied in some cases by bubbles of CO_2_ and in others by apparent inactivity.

### 2.2. Sampling Strategy and Samples Collection

**Figure 1:**
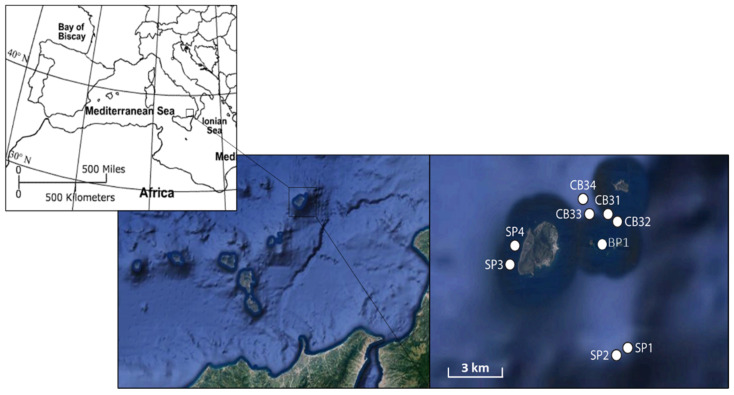
Study area and location of sampling sites within each sub-area. SP indicate samples collected at the Secca dei Pesci, CB3 indicated samples collected off the southwestern coast of the islet of Basiluzzo and BP is the Black Point site at the Archipelago of Dattilo, Panarelle, Lisca Bianca, Bottaro and Lisca Nera (Color maps are generated from Google Earth: Map data ©2021 Google Earth data SIO, NOAA, US Navy, NGA, GEBCO, Landsat/Copernicus).

Sampling activities in the Panarea area were carried out during the ISPRA “ORBS PANA_15” scientific cruise on board of the R/V Astrea using a 25 L Van Veen grab, in June 2015. Two different sub-areas were investigated: one off the southwestern coast of the islet of Basiluzzo (named CB3, with four sampling stations: CB3-1, CB3-2, CB3-3, and CB3-4) and located at ca. 77 m of depth, and one at the southeastern sector of the Panarea Volcanic Complex, in the shallow region (ca. 40 m of depth) known as “Secca dei Pesci” (named SP, with two sampling stations: SP1 and SP2) (Figure 1). Both sub-areas were first investigated by means of multi-beam prospection, to identify venting sites [29]. In the Basiluzzo sub-area, a benthic chamber was deployed for 6 h in 3 different sites (depth range 74–81 m) characterized by potential venting activity to carry out measurements of temperature (T), DIC, metals, and H+ over time. In the Secca dei Pesci sub-area, the pH and T were measured through a CTD cast along the water column, which allowed to identify one vent site (SP1, close to an intense hydrothermal active vent with strong CO_2_ emission, i.e., ppm 1400) and one non-venting station (SP2). Two additional sampling stations were selected far from the SP sub-area at comparable depths, named as SP3 and SP4, with no vents activity. Thus, SP2-SP4 were considered as control stations (hereafter inactive vents). Similarly, at the CB3 sub-area, two stations were characterized by the presence of CO_2_ emission vents (CB3-2 and CB3-3), while in the other two (CB3-1 and CB3-4), no activity was recorded; thus, they were used as the control (e.g., inactive vents). Additional samples were collected at a shallower site named Black Point (BP1, at 16 m of depth), characterized by strong emission, and located inside a small archipelago consisting of five islets (Figure 1). At those sites characterized by CO_2_ emission, the pH values ranged between 5.80 and 6.07 (at SP1 and CB3-3, respectively), while at the control sites, the pH varied between 7.10 and 8.17, at SP2 and CB3-4, respectively.

Sediment samples for the analysis of the sediment organic matter (0–1 cm), meiofauna, and macrofauna (both collected down to a depth of 10 cm) were sampled in three independent replicates in each site and stored at −20 °C until the analyses in the laboratory. To cope with the possible bias raised by using the Van Veen grab, which may produce leaking of interstitial water during recovery, we collected samples only from deployments in which the grab was completely watertight, by visual inspection. Moreover, the grab was equipped with rubber flaps over screened opening to prevent washout [30]. Sediment sub-samples for the subsequent analyses of organic matter (OM) and meiofauna were collected from three independent deployments of the grab by means of plexiglass corers (internal diameter 3.6 cm). As the grain size at different sites was characterized by large sediment fraction (i.e., rodolith beds) making the sieving of samples on board difficult, through 20 and 500 μm sieve, for meio- and macrofauna, respectively, the whole samples were kept frozen (see review in [31]).

### 2.3. Biochemical Composition of Sedimentary Organic Matter

Data on the biochemical composition of sedimentary organic matter are reported in Tangherlini et al. [32].

### 2.4. Meio- and Macrofauna Samples Processing

Each sediment sample for meiofauna was treated with ultrasound (for 1 min, three times, with 30 s intervals) to detach organisms from the grain particle surface and then, sieved through a 500 µm (no large meiofaunal organisms were retained in this first sieve, at a visual check) and a 20 µm mesh net to retain the smallest organisms. For samples collected in CB3 and BP1, the fraction remaining on the latter sieve was re-suspended and centrifuged three times with Ludox HS40 diluted with water to a final density of 1.18 gcm^−3^ [30]. For samples collected in SP, meiofauna were extracted using the decantation method [33]. Once the extraction was completed, the quality check of the extraction efficiency was 100% in both approaches (verified through sample inspection after three centrifugations in the first approach, and after 10 baths in the second). All specimens from three independent replicates per sampling site were counted and sorted by taxa, under a stereomicroscope after staining with Rose Bengal (0.5 gL^−1^) and stored in ethanol at 70%. Meiofaunal biomass was assessed by bio-volumetric measurements of all retrieved specimens. Nematode biomass was calculated from their biovolume, using the Andrassy (1956) formula (V = L × W^2^ × 0.063 × 10^−5^, in which body length, L, and width, W, are expressed in µm). Body volumes of all other taxa were derived from measurements of body length (L, in mm) and width (W, in mm), using the formula V = L × W^2^ × C, where C is a dimensionless factor (specific for each meiofaunal taxon) used to convert L × W^2^ to body volume, according to models relating body dimensions and volume [34]. Each body volume was multiplied by an average density of 1.13 gcm^−3^ to obtain the biomass (µg dry weight: µg wet weight =0.25; ref. [35] and the carbon content was considered to be 40% of the dry weight [34]. Abundance data were expressed as individuals/10 cm^2^ [34].

In the laboratory, macrofauna samples were thawed, sieved through a 500 μm sieve, and first separated in higher taxonomical groups (Polychaeta, Bivalvia, Gastropoda, Amphipoda, Isopoda, etc.). Then, the extracted organisms were identified to the lowest taxonomic level, counted and weighed, and standardized to m^2^, to obtain the abundance (number of individuals/m^2^) and biomass (mg WW/m^2^) data.

### 2.5. Data Analysis

Both univariate and multivariate analyses were based on a two-way sampling design: “vents” as a fixed factor, with two levels, active vs. inactive (as control), and “depth”, fixed, with two levels, shallow (SP stations at depths between 37 and 41 m) vs. deep sites (CB3 stations at depths ranging from 74 m to 81 m), crossed within each other. Since the BP site had no replicates, it was removed from statistical analyses, to maintain a crossed design, and represented only in descriptive analyses. Changes in abundance, biomass, and diversity (number of meiofaunal taxa and of macrofaunal species richness) were tested by univariate PERMANOVA [36], based on the Euclidean resemblance matrix of log(x + 1) transformed abundance, biomass, and untransformed diversity data, respectively. Abundance, biomass, and diversity data were also visualized by boxplots.

Changes in community composition were analyzed through PERMANOVA test run on the Bray–Curtis resemblance matrix of square root for meiofauna, and log(x + 1)-transformed for macrofauna abundance data, and the pair-wise tests were carried out for the interaction factor, if significant in the main test. On the same matrix, a CAP analysis [37] was run on the factor found to be significant by PERMANOVA, to visualize the differences in meio- and macrofaunal composition among sampling sites. To assess the percentage of similarity/dissimilarity in the taxonomic composition of meio- and macrofauna at active vs. inactive sites and at shallow vs. deep sites, a SIMPER analysis was carried out. In the case of meiofauna, SIMPER analysis was performed with and without nematodes once they tended to also dominate in stressful environmental conditions [38]. A shade plot was also used to represent the total abundance by higher taxonomical group of macrofauna (class or order) in active vs. inactive sites [38].

Finally, a distance-based linear model (DistLM, [39]) was run on both meio- and macrofaunal composition matrix and the variables obtained by the analysis of the sedimentary organic matter, and the total meiofauna and nematodes abundance as additional variables for the model run on macrofaunal abundance matrix. In both cases, DistLM models were performed by using a stepwise selection procedure and adjusted R^2^ as the selection criterion. Environmental variables were first tested for normality and collinearity with Draftsman’s plot. Non-normal distributed variables were log(x + 1) transformed, while redundant variables (at ρ > 0.7) were eliminated from the analysis. Specifically, as the Draftsman’s plot showed collinearity between biopolymeric carbon fraction (BPC) and protein (PRT) and carbohydrate (CHO) content, at 0.89 and 0.91, respectively, BPC was eliminated from the model running.

All statistical analyses were carried out by using PRIMER7 and PERMANOVA+ software [36,40].

## 3. Results

### 3.1. Abundance and Biomass of Meiofauna

**Figure 2:**
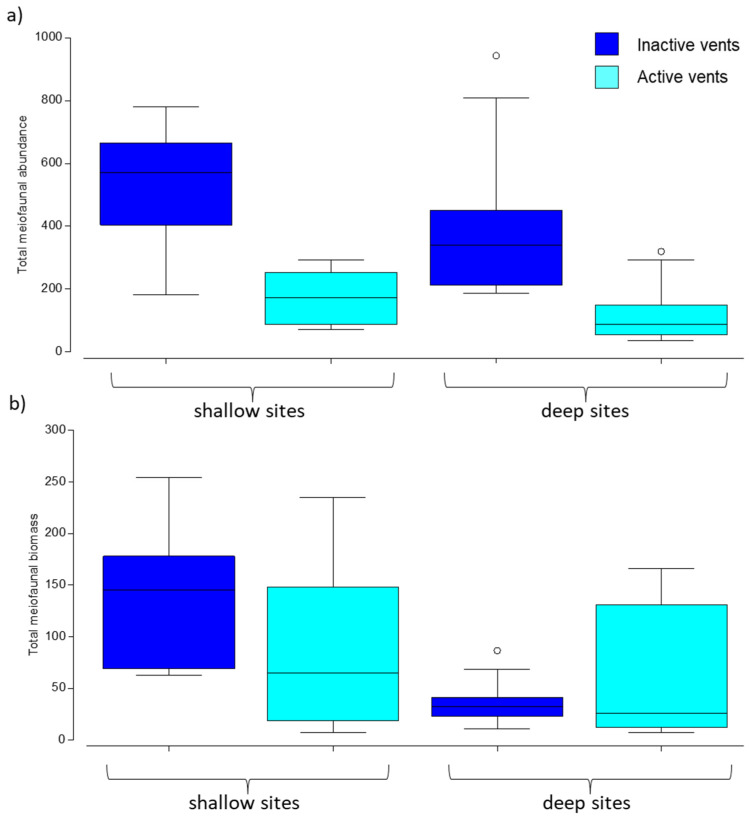
Boxplots of (**a**) abundance (N individuals/10 cm^2^) and (**b**) biomass (μgC/10 cm^2^) of meiofauna in active (shallow and deep) and inactive (shallow and deep) sites. Colors indicate active (light blue) and inactive (dark blue) vents and x-axis defines shallow vs. deep sites. Lines in the box represent the median. Circles are outliers (observation points that is distant from other observations).

The meiofaunal abundance decreased significantly from inactive to active sites, with significant differences at both shallow and deep locations (Table 1a,b, Figure 2a). Conversely, the meiofaunal biomass showed a nonlinear trend, with higher biomass values at inactive vents only at shallow sites (Figure 2b). Consistently, the meiofaunal biomass varied significantly only for the factor depth (Table 1a).

### 3.2. Abundance and Biomass of Macrofauna

**Figure 3:**
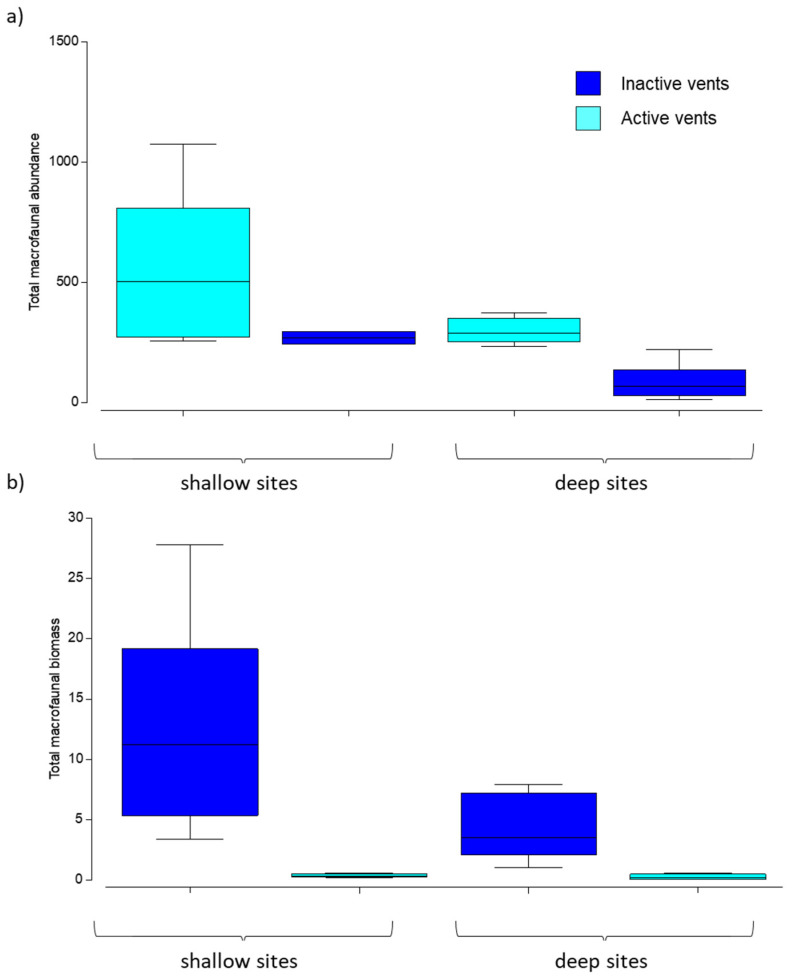
Boxplots of (**a**) abundance (N individuals/m^2^), and (**b**) biomass (g WW/m^2^) of macrofauna in in active (shallow and deep) and inactive (shallow and deep) sites. Colors indicate active (light blue) and inactive (dark blue) vents and x-axis defines shallow vs. deep sites. Lines in the box represent the median.

Higher abundance and biomass values were found in inactive sites compared to those characterized by CO_2_ emission (Table 2a and Figure 3a,b). Abundance and biomass values significantly decreased between active vs. inactive sites at the deep sites and for both abundance and biomass and at the shallow ones only for biomass (Table 2b).

### 3.3. Meio- and Macrofaunal Diversity and Community Composition

Overall, 18 meiofaunal taxa were reported in the present study. Meiofaunal diversity, as number of meiofaunal higher taxon, ranged from 7 (CB3-4, inactive) to 16 (SP2, inactive), respectively. Nematodes were the dominant taxon (on average 74%), followed by copepods (12%), ostracods (4%), polychaetes and halacarids (3%), and cladocerans (1%). All other taxa were rare.

**Figure 4:**
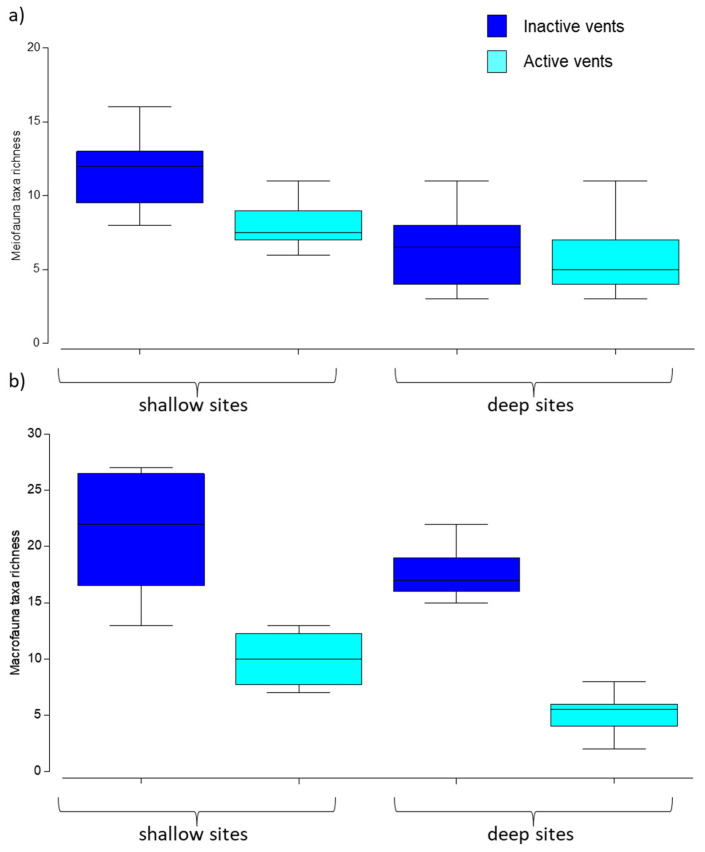
Boxplots of meiofaunal higher taxa richness (**a**) and macrofauna species richness (**b**) recorded in active (shallow and deep) and inactive (shallow and deep) sites. Colors indicate active (light blue) and inactive (dark blue) vents and x-axis defines shallow vs. deep sites. Lines in the box represent the median.

The meiofaunal diversity was higher, though not significant, at inactive vents, while it differed significantly between shallow vs. deep sites (Table 3a), with the highest values at shallow sites (Figure 4a).

The macrofaunal diversity varied significantly for both the factors investigated (Table 3a), being greater at inactive vs. active sites at both the depths explored (Table 3b and Figure 4b). Differences in species richness between inactive vs. active sites were particularly evident at the deeper stations.

Changes in community composition were evident for both meio- and macrofauna, when comparing active vs. inactive sites. The PERMANOVA test showed that both meio- and macrofaunal community significantly changed in relation to the presence of CO_2_ emission (Table 4a). The pairwise comparisons indicated significant differences between samples collected at inactive vs. active sites only at shallow sites for meiofauna and at both depths for macrofauna (Table 4b).

**Figure 5:**
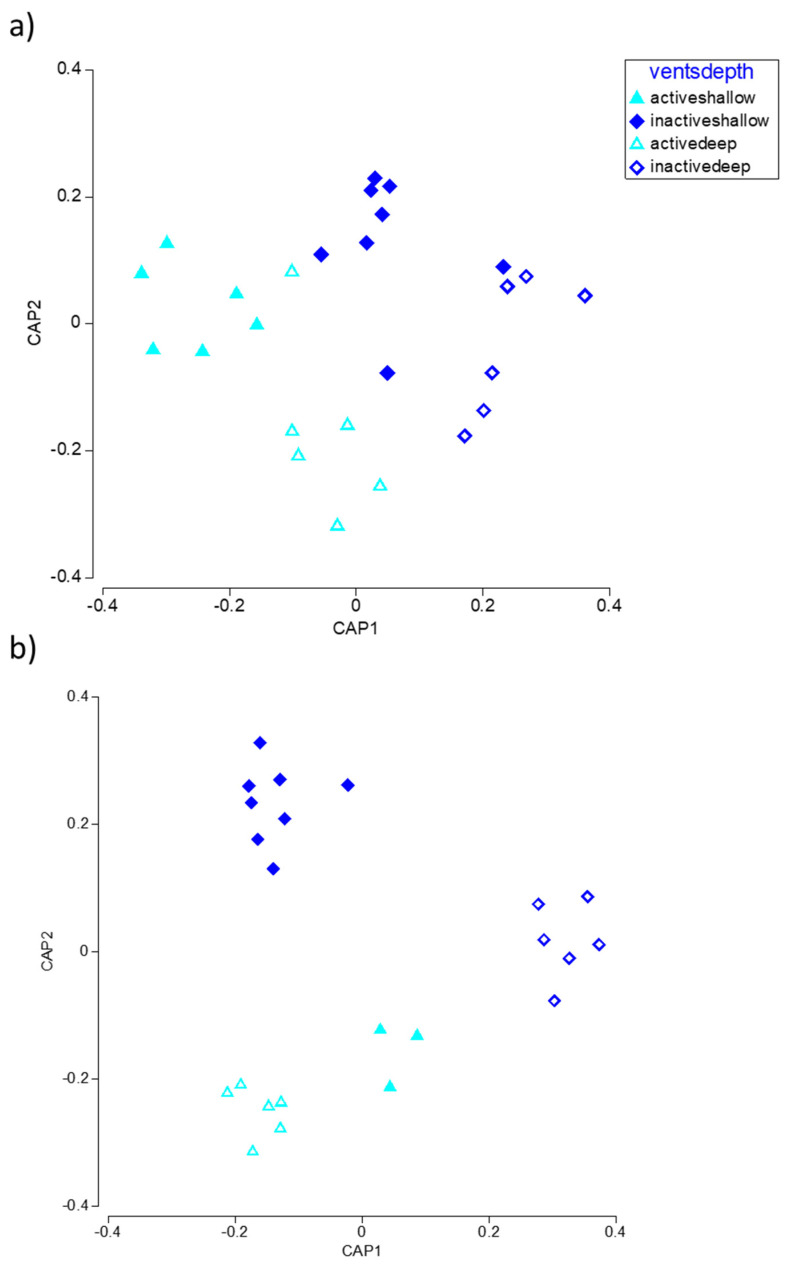
CAP plot of the meio- (**a**) and macrofauna (**b**) composition of the samples collected at active vs. inactive vents and at shallow vs. deep sites around Panarea island.

The CAP analysis revealed a good separation of meiofaunal assemblages at inactive vs. active vents (Figure 5a), while a clear segregation of samples also by depth occurred for macrofauna, especially for samples collected at inactive vents (Figure 5b).

The SIMPER analysis identified the meiofaunal taxa that mostly contributed to the similarity/dissimilarity between inactive vs. active sites at each depth (Table S1a,b in SOM). Considering all taxa, nematodes and copepods were present in all sites regardless the vent type (i.e., active vs. inactive) and depth (shallow vs. deep, Table S1a,b). At both shallow and deep sites, ostracods and polychaetes contributed to dissimilarity among samples together with nematodes and copepods (Table S1b), being more abundant at inactive sites. Considering only rare taxa (Table S1c,d), cladocerans and halacaroids were responsible for the dissimilarity, being more abundant at the active sites (Table S1c), while tardigrades and priapulids were dominant in the inactive sites.

As far as the macrofauna is concerned, while syllid, onuphid, and eunicid polychaetes seemed to be the most sensitive taxon to CO_2_ emission, together with different bivalve species, being abundant only at inactive sites, oligochaetes appeared to be the most tolerant group, dominating at active vents (Table S2a). Differences in the taxa contributing to dissimilarity were evident at both depths (Table S2b), with *Caprella* sp. and Syllidae as dominant at active and inactive vents, respectively, at shallow depths, and Onuphidae and Oenonidae, and oligochaetes abundant at active and inactive vents, respectively, at deep sites.

**Figure 6:**
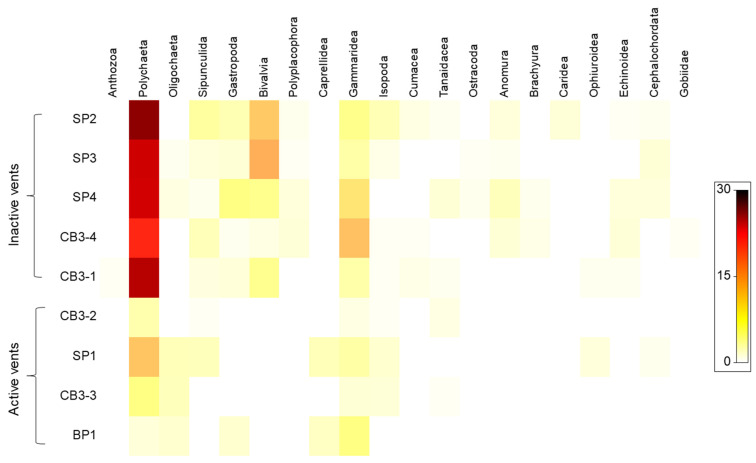
Shade plots on macrofaunal composition recorded at each sampling station. Colors indicate taxon abundance as in the legend.

A shade plot (Figure 6) based on the abundance of the macrofaunal classes/orders found at each station shows a decrease in polychaetes and disappearance of mollusks, sipunculids, crustaceans (except for gammarids and caprellids), echinoderms, and chordates at active vents.

### 3.4. Correlation of Meio- and Macrofaunal Communities and Organic Matter Quality and Quantity

Concerning meiofauna, the results of the model showed that four variables explained the observed pattern of changes in community composition; however, after fitting the first two variables, the *p*-value associated to add lipids (LIP) to the model was not statistically significant and quite large (*p* = 0.321). The two significant variables (protein concentration-PRT and total phytopigments-TPH) explained 30% of the total variance (Table 5).

Six variables explained the total variation for macrofauna (Table 5). The first four variables (TPH, PRT, percentage -%PRT and carbohydrate percentage -%CHO), together explained 33% of the variation in community structure, and subsequent variables added little to the total variation (about 4–6% each) and were not significant.

## 4. Discussion

While the biodiversity, adaptation, and response to fluid emissions have been widely investigated in deep-sea hydrothermal vents, the response of meio- and macrofauna to CO_2_ volcanic emission at shallower (<100 m) depths has received much less attention. Here, we report the changes observed for meiofaunal and macrofaunal assemblages at active and inactive vent sites from different depths.

### 4.1. Changes in Meio- and Macrobenthic Abundance and Biomass Due to CO_2_ Emission

A drastic decrease in the abundance of benthic fauna moving towards active vents is expected due to the extreme chemical and physical conditions encountered around these sites [41]. Overall, we observed a decrease in meio- and macrofaunal abundance near active hydrothermal vents, where the CO_2_ concentrations increased up to 1400 ppm. At the same time, the impact in terms of biomass was significant only for macrofauna. A previous study conducted in the same area and investigating the impact of fluid emissions on benthic prokaryotes [32] revealed that both prokaryotes and viruses remained consistently higher in sediments influenced by CO_2_ discharges than in inactive sites. These results suggest that acidification due to CO_2_ leakage can differently impact the diverse components of the benthic system.

Previous studies have also shown a significant negative effect on the abundance and growth of several marine taxa (e.g., oysters and mussels, warm-water corals, and cold-water coralline algae), with a mean reduction of 15% for abundance, and of 11% for growth rates [42].

For meiofauna, we observed that water acidification seems to affect the abundance but not on biomass. This finding may suggest that the emissions had negligible effects on the growth rates of meiofaunal taxa or that large-size meiofaunal taxa were not more sensitive to acidification than small-sized taxa. Despite the extreme conditions close to the vent emissions, the values of abundance reported here are higher than those reported in other coastal vents, where the meiofaunal abundance was 5–10 times lower than in the present study [8]. Among the emission vent sites, the highest abundance is observed in the shallowest site, revealing the importance of local environmental conditions in influencing meiofaunal assemblages.

The decrease in both macrofaunal abundance and biomass close to the vent sites could be related to changes in the species growth rate, particularly mollusks, which are the second most dominant group in soft-bottom macrofauna samples. There is an increased metabolic cost of coping with hypercapnia, which also leads to dwarfism phenomena [43] or an increased susceptibility to predation [44].

### 4.2. Effects of Acidification on Meio- and Macrofauna Community Composition and Diversity

The available literature on the benthic community responses to low pH suggests that one of the main consequences is a shift in community structure (see [44] and references cited therein, [12,45,46]).

In the present study, we reported significant differences at the community level in both meiofauna and macrofauna between inactive and active vents.

The negative effects of acidification on meiofaunal diversity have been documented in previous investigations carried out in in situ conditions [8]. At the same time, experimental studies conducted in mesocosms reported contrasting results [8,47,48,49,50] of the effects of low pH on the abundance of meiofaunal taxa, with some experiments showing decreased abundances of calcifying taxa (i.e., Gastotricha, [47]) and unaffected abundances of dominant taxa, and others showing equal or higher abundances for dominant taxa (i.e., Harpacticoida and Polychaeta abundances without variations and Nematoda, Ostracoda, Turbellaria, and Tardigrada exhibiting their highest densities in low-pH treatments.

Most of the available studies aimed to assess the effects of acidification on meiofauna are indeed based on laboratory experiments and focused on the dominant taxa such as nematodes and copepods [51,52].

The findings of the present investigation indicate that at high CO_2_ concentrations, the meiofaunal diversity and community composition change as a result of the different sensitivity of the various taxa.

Nematodes, followed by copepods, are generally the key component of meiofaunal assemblages inhabiting sediments around the vents [8,53,54,55,56,57]. Accordingly, in the present study, these two taxa dominated at all sites, including in those influenced by vent emissions. However, most of the changes in taxa/species composition occur amongst the rare taxa [20,58,59]. Here, we report that, among rare taxa, kinorhynchs and turbellarians are the most sensitive to the acidification and disappeared in all sites characterized by CO_2_ emissions. Bivalves and priapulids showed a similar sensitivity to CO_2_ emissions and were present only in inactive sites. We cannot exclude that the different vent emissions along with the local environmental conditions and depth could influence the presence/absence of some other taxa. At the same time, we cannot discount the potential influence of indirect effects linked to changes in food availability as well as top-down effects due to meiofaunal predation.

Furthermore, we report that halacarids and ostracods increased their abundance in sediments characterized by CO_2_ emissions, and this could be a direct response to the large availability of organic matter along with an increase of prokaryotic abundance [32].

The high meiofaunal variability observed at some sites in active vents is likely associated with the complexity of hydrothermal vent fields, small-scale habitats that change at scales of centimeters, influenced by topographical variables. Changes in composition along the vent transition zone may be abrupt or gradual, vary according to vent characteristics, such as intensity, size, and regional context [57,60]. In this sense, additional studies are recommended to investigate the natural variability in structure by in situ time series of vents and associated fauna [61].

Macrofauna also showed a clear community shift from active to inactive vent sites. The taxa that most contributed to the discrimination between active and inactive sites were syllid polychaetes, sipunculids, gastropods, and bivalves. A gradient of vulnerabilities to exposure to low-pH conditions have also been previously reported for mollusks > arthropods > annelids [12,45,46,52,62,63]. Calcifying organisms, such as mollusks, are proposed as the most vulnerable taxa to low pH, especially at larval and juvenile stages [64,65,66,67]. Moreover, they generally possess poorer regulatory abilities [68,69,70,71,72]. In our study, evident effects were also observed for the sipunculid *Phascolion (Phascolion) strombus strombus*, which preferentially lives inside the shells of gastropods (here *Bittium reticulatum*) acting as drivers of sipunculid distribution [73].

At the species level, inactive vents are dominated by the Eunicid polychaete *Lysidice unicornis*, a species characteristic of rodolith beds [74], which disappeared in fluid vent areas [27]. Low pH sites, and especially the “smoking land”, were dominated by the red algae *Peyssonellia* sp. This alga creates a secondary substratum for the settlement of encrusting and sessile organisms, such as hydrozoans, which could explain the abundance of caprellids, frequently associated to hydrozoans [75] at the vent sites.

Several macrofaunal species of shallow-water vents are typical of organic-rich/reducing sediments. Here, sensitive macrofaunal species disappeared almost completely, while opportunistic species such capitellid polychaetes and oligochaetes increased in abundance [55,56,57,76]. Oligochaetes are indicators of environmental quality and have a high tolerance to high organic concentrations, including pollutants. Moreover, they can tolerate environments characterized by low pH, varying temperatures and salinity, and oxygen deficiency [77]. As a result, these organisms tolerate high CO_2_ emissions [78].

The shallow-vent fauna is usually a sub-set of the surrounding community that contains a limited number of species capable of withstanding the vent environment (e.g., [54,57]). At active vent sites, we also found different abundant lyssianassyd and *Leucothoe* sp., which are found to be very abundant in extreme conditions such as deep-sea hydrothermal vents [79]. Some of the dominant polychaete taxa showed relatively high and uniform abundances at active and controls sites, such as Hesionidae and *Pisione* sp., while *Lumbrineris* was almost exclusively present at vents sites, demonstrating some degree of tolerance to OA. Conversely *Lysidice* spp. occurred only at inactive sites, suggesting that polychaete tolerance (or vulnerability) to OA is highly species-specific [80]. Amphipods of the genus *Caprella* only occurred at the vent sites, as also observed in other studies [81].

The emission areas are therefore drivers that shape the structure of macrofaunal assemblages [82] and their diversity. Even a small decrease in pH levels can lead to relevant changes in benthic systems with altered community structure and composition of macrofaunal communities [83].

### 4.3. Ocean Acidification Changes in Food Availability as Drivers of Community Composition Shifts

Both DistLM models for meiofauna and macrofauna showed that food availability (protein and total phytopigment concentrations) were crucial driving forces of community composition. Sediments surrounding the sites with vent emissions exhibit a relative high concentration of the most labile compounds of the organic matter, especially proteins, and a preferential accumulation of phytopigments. The larger availability of high-quality food sources could explain the higher abundance and diversity of meiofauna generally observed in the present study. At the same time, the increased food availability can offset reductions in calcification and growth associated with acidification in corals and mussels [84,85,86,87] and thus support moderate abundance values also at vent sites, including the importance of herbivores, sustained by the high primary biomass (as indicated by the total phytopigment concentration), which can counterbalance the impact of acidification [88,89].

## 5. Conclusions

Our results show a clear impact of CO_2_ emissions from shallow hydrothermal vents of the Aeolian Islands on both meio- and macrofaunal assemblages at both shallow and deep sites. However, while acidification impacted directly on highly sensitive meio- and macrofaunal taxa, the decrease in other taxa may be caused by the effect of vent emissions on food availability. Kinorhynchs and turbellarians, for instance, disappeared at low-pH sites. Other cascade effects may be due to altered top-down interactions. Among macrofauna, calcifying organisms, especially gastropods, appeared more sensitive than amphipods and brittle stars to acidification. Finally, the polychaetes response to low pH varied from species to species.

The results of this study indicate that the effects of fluid vents and related acidification cannot be easily predicted as the response of the different components of the benthic system (even within the same size class) may vary drastically. Further studies are needed to fully elucidate the complex physiological mechanisms allowing a different tolerance of the benthic species and the cascade ecological effects due to the disappearance of some taxa and the altered food availability.

## Figures and Tables

**Table 1:** Results of the univariate PERMANOVA main (a) and pairwise tests (b) for term ‘Vents × Depth’ for pairs of levels of factor ‘Vents’, carried out on the abundance and biomass of meiofauna (in (a)), and in (b) only for abundance) collected at active vs. inactive sites at the two depth ranges (shallow vs. deep). df = degrees of freedom; MS = mean square; F = statistic F; t = statistic t for pairwise comparisons.

**(a)**			**Abundance**		**Biomass**	
	**Source**	**df**	**MS**	**F**	**MS**	**F**
	**Vents**	1	9.05	22.87 ***	0.07	0.18 ^ns^
	**Depth**	1	0.45	1.15 ^ns^	4.42	11.15 **
	**Vents × Depth**	1	0.01	0.02 ^ns^	0.17	0.44 ^ns^
	**Residuals**	19	0.4		0.4	
	**Total**	22				
**(b)**		**Within level ‘shallow’ of factor ‘Depth’ for abundance**				
	**Groups**		t			
	**Active vs. Inactive**		3.76 *			
		**Within level ‘deep’ of factor ‘Depth’ for abundance**				
	**Groups**		t			
	**Active vs. Inactive**		3.22 *			

*** indicates *p* < 0.001; ** indicates *p* < 0.01; * indicates *p* < 0.05; ^ns^ means not significant differences.

**Table 2:** Results of the univariate PERMANOVA main (a) and pairwise tests (b) for term ‘Vents × Depth’ for pairs of levels of factor ‘Vents, carried out on the abundance and biomass of macrofauna collected at active vs. inactive sites and at shallow vs. deep sites. Df = degrees of freedom; MS = mean square; Pseudo-F = statistic F; t = statistic t for pairwise comparisons.

**(a)**			**Abundance**		**Biomass**	
	**Source**	**df**	**MS**	**F**	**MS**	**F**
	**Vents**	1	262,080	6.25 *	276.88	7.70 *
	**Depth**	1	206,290	4.92 *	80.89	2.25 ^ns^
	**Vents × Depth**	1	7410.1	0.18 ^ns^	74.07	2.06 ^ns^
	**Residuals**	18	41,912		35.94	
	**Total**	21				
**(b)**		**Within level ‘shallow’ of factor ‘depth’**				
	**Groups**		t		t	
	**Active vs. Inactive**		1.24 ^ns^		1.79 *	
		**Within level ‘deep’ of factor ‘depth’**				
	**Groups**		t		t	
	**Active vs. Inactive**		5.38 **		3.40 **	

** indicates *p* < 0.01; * indicates *p* < 0.05; ^ns^ means not significant differences.

**Table 3:** Results of the univariate PERMANOVA main (a) and pairwise tests (b) for term ‘Vents × Depth’ for pairs of levels of factor ‘Vents’, carried out on the richness of higher taxa for meiofauna and species richness for macrofauna collected at active vs. inactive sites and at shallow vs. deep sites. df = degrees of freedom; MS = mean square; Pseudo-F = statistic F; t = statistic t for pairwise comparisons.

**(a)**		**Meiofaunal Richness of Higher Taxa**			**Macrofaunal Species Richness**		
	**Source**	**df**	**MS**	**F**	**df**	**MS**	**F**
	**Vents**	1	19.79	2.60 ^ns^	1	712.5	45.18 ***
	**Depth**	1	73.44	9.63 **	1	89.48	5.67 *
	**Vents × Depth**	1	8.7	1.14 ^ns^	1	1.97	0.12 ^ns^
	**Residuals**	19	7.63		18	15.77	
	**Total**	22			21		
**(b)**		**Within level ‘shallow’ of factor ‘depth’**					
	**Groups**					t	
	**Active vs. Inactive**		_			3.17 *	
		**Within level ‘deep’ of factor ‘depth’**					
	**Groups**					t	
	**Active vs. Inactive**		_			9.48 **	

*** indicates *p* < 0.001; ** indicates *p* < 0.01; * indicates *p* < 0.05; ^ns^ means not significant differences.

**Table 4:** Results of the multivariate PERMANOVA main (a) and pairwise tests (b) for term ‘Vents × Depth’ for pairs of levels of factor ‘vents activity’, carried out on the meio- and macrofaunal community composition of samples collected at active vs. inactive sites. df = degrees of freedom; MS = mean square; Pseudo-F = statistic F; t = statistic t for pairwise comparisons.

**(a)**		**Meiofauna**			**Macrofauna**		
	**Source**	**df**	**MS**	**Pseudo-F**	**df**	**MS**	**Pseudo-F**
	**Vents**	1	3819.8	5.95 ***	1	10,354	5.28 ***
	**Depth**	1	5665.4	8.83 ***	1	6838	3.49 ***
	**Vents × Depth**	1	602.05	0.94 ^ns^	1	5186.3	2.64 **
	**Residuals**	19	641.78		18	1962.1	
	**Total**	22			21		
**(b)**			**Within level ‘shallow’ of factor ‘depth’**				
	**Groups**		t			t	
	**Inactive vs. Active**		2.49 **			2.16 **	
			**Within level ‘deep’ of factor ‘depth’**				
	**Groups**		t			t	
	**Inactive vs. Active**		1.41 ^ns^			2.06 **	

** = *p* < 0.01, *** = *p* < 0.001, ^ns^ means not significant differences.

**Table 5:** Results of the DistLM models run on meiofauna and macrofauna resemblance matrix and potential drivers of change. Adj R^2^ = adjusted R^2^; Pseudo-F = statistic F; *p* = probability level; Prop. = proportional percentage of variance explained by the explanatory variable; Cumul. = cumulative percentage of variance explained by the explanatory variables; res.df = residual degrees of freedom; PRT and %PRT = proteins concentration and percentage, respectively, in sedimentary organic matter (SOM); LIP = lipids concentration in SOM; CHO and %CHO: carbohydrates concentration and percentage, respectively, in SOM; TPH: total phytopigments.

**Meiofauna**						
**Variable**	**Adj R^2^**	**Pseudo-F**	***p***	**Prop.**	**Cumul.**	**res.df**
PRT	0.13	4.76	0.004	0.17	0.17	24
TPH	0.24	4.54	0.005	0.14	0.30	23
LIP	0.25	1.20	0.321	0.04	0.34	22
%PRT	0.28	1.99	0.088	0.06	0.40	21
**Macrofauna**						
**Variable**	**Adj R^2^**	**Pseudo-F**	***p***	**Prop.**	**Cumul.**	**res.df**
TPH	0.07	2.57	0.004	0.11	0.11	21
PRT	0.11	2.02	0.023	0.08	0.19	20
%PRT	0.15	1.87	0.031	0.07	0.26	19
%CHO	0.18	1.84	0.029	0.07	0.33	18
CHO	0.21	1.59	0.085	0.06	0.39	17
Nematoda	0.22	1.25	0.276	0.04	0.43	16

## Data Availability

The data presented in this study are available on reasonable request from the corresponding author.

## Supplementary Materials

The following supporting information can be downloaded at: https://www.mdpi.com/article/10.3390/biology11020321/s1, Table S1: Results of the SIMPER analysis with the taxa contributing to similarity (a–c) and dissimilarity (b,d) considering active vs. inactive vents and shallow vs. deep sites for meiofauna, for all taxa (a,b) and only for rare taxa (c,d); Table S2: Results of the SIMPER analysis with the taxa contributing to similarity (a) and dissimilarity (b) considering active vs. inactive vents and shallow vs. deep sites for macrofauna.
