# Supplementary material for: Effects of Local Acidification on Benthic Communities at Shallow Hydrothermal Vents of the Aeolian Islands (Southern Tyrrhenian, Mediterranean Sea)
Source: Biology (Basel). 2022 Feb 17;11(2):321. doi: 10.3390/biology11020321 (PMC8868750; doi:10.3390/biology11020321)
Supplement: Supplementary file 1 [file biology-11-00321-s001.zip › biology-1516996-supplementary.pdf]

Supplementary Material to

Effects of local acidification on benthic communities at shallow hydrothermal vents of the Aeolian Islands (Southern Tyrrhenian, Mediterranean Sea)

Fanelli E.*[1,2], Di Giacomo S.[1], Gambi C.[1], Bianchelli S.[1], Da Ros Z.[1], Tangherlini M.[2], Andaloro F.[2], Romeo T.[2], Corinaldesi C.[3], Danovaro R.[1,2]

1 Department of Life and Environmental Sciences, Polytechnic University of Marche, Via Brecce Bianche 60131 Ancona (Italy)
2 Stazione Zoologica di Napoli Anton Dohrn, Villa Comunale 80100 Naples (Italy)
3 Department of Materials, Environmental Sciences and Urban Planning (SIMAU), Polytechnic University of Marche, Via Brecce Bianche 60131 Ancona (Italy)
* corresponding author, E. Fanelli: e.fanelli@univpm.it

Table S1. Results of the SIMPER analysis with the taxa contributing to similarity (a-c) and dissimilarity (b-d) considering active *vs.* inactive vents and shallow *vs.* deep sites for meiofauna, for all taxa (a-b) and only for rare taxa (c-d). Cut-off for low contribution at 60%. Avg. Ab=average abundance; Avg. Sim=average similarity; Contrib %= percentage of variance explained by the explanatory variables; Cum %= cumulative percentage of variance explained by the explanatory variables; Avg. Diss=average dissimilarity.

a)

**Group active shallow**		**Av.Sim: 68.86**		
**Taxon**	**Av.Abund**	**Av.Sim**	**Contrib%**	**Cum.%**
Nematoda	8.12	18.72	27.19	27.19
Copepoda	6.37	17.33	25.16	52.36
Ostracoda	4.23	11.37	16.52	68.87
**Group inactive shallow**		**Av.Sim: 73.55**		
Nematoda	19.78	36.77	49.99	49.99
Copepoda	7.65	11.58	15.74	65.74
**Group active deep**		**Av.Sim: 58.04**		
Nematoda	9.11	41.57	71.63	71.63
**Group inactive deep**		**Av.Sim: 72.45**		
Nematoda	18.96	55.54	76.66	76.66

b)

**Shallow sites: active *vs.* inactive vents**			**Av.Diss = 39.07**		
	**Active**	**Inactive**			
**Taxon**	**Av.Abund**	**Av.Abund**	**Av.Diss**	**Contrib%**	**Cum.%**
Nematoda	8.12	19.78	15.31	39.18	39.18
Copepoda	6.37	7.65	4.09	10.46	49.63
Ostracoda	4.23	3.55	2.37	6.07	55.7
Polychaeta	2.11	3.08	2.2	5.63	61.33
**Deep sites: active *vs.* inactive vents**			**Av.Diss = 44.85**		
	**Active**	**Inactive**			
Nematoda	9.11	18.96	21.64	48.25	48.25
Copepoda	3.12	2.83	4.6	10.25	58.5
Ostracoda	1.8	0.81	2.88	6.42	64.92

c)

**Group active shallow**		**Av.Sim: 63.09**		
**taxon**	**Av.Abund**	**Av.Sim**	**Contrib%**	**Cum.%**
Ostracoda	4.23	25.98	41.18	41.18
Acarina	3.45	21.89	34.69	75.87
**Group inactive shallow**		**Av.Sim: 57.29**		
Ostracoda	3.55	14.18	24.75	24.75
Acarina	2.93	12.18	21.26	46.01
Tardigrada	1.85	9.44	16.47	62.48
**Group active deep**		**Av.Sim: 29.68**		
Ostracoda	1.8	21.59	72.73	72.73
**Group inactive deep**		**Av.Sim: 34.53**		
Larva Priapulida	1.07	17.23	49.89	49.89
Tardigrada	1.19	7.74	22.42	72.32

d)

**Shallow sites: active vs. inactive vents**			**Av.Diss = 44.42**		
	**Active**	**Inactive**			
**Taxon**	**Av.Abund**	**Av.Abund**	**Av.Diss**	**Contrib%**	**Cum.%**
Ostracoda	4.23	3.55	6.18	13.91	13.91
Acarina	3.45	2.93	4.75	10.7	24.61
Tardigrada	0.82	1.85	4.49	10.1	34.71
Larva Gasteropoda	0.37	1.39	4.12	9.28	43.99
Cladocera	2.34	1.66	3.87	8.72	52.71
Amphipoda	0.9	1.02	3.31	7.46	60.17
**Deep sites: active vs. inactive vents**			**Av.Diss = 77.01**		
	**Active**	**Inactive**			
Species	Av.Abund	Av.Abund	Av.Diss	Contrib%	Cum.%
Ostracoda	1.8	0.81	14.88	19.32	19.32
Larva Priapulida	0.17	1.07	13	16.88	36.2
Tardigrada	0	1.19	10.99	14.27	50.48
Acarina	0.64	0.66	8.27	10.74	61.22

Table S2. Results of the SIMPER analysis with the taxa contributing to similarity (a) and dissimilarity (b) considering active *vs.* inactive vents and shallow *vs.* deep sites for macrofauna. Cut-off for low contribution at 60%. Av. Ab=average abundance; Av. Sim=average similarity; Contrib %= percentage of variance explained by the explanatory variables; Cum %= cumulative percentage of variance explained by the explanatory variables; Avg. Diss=average dissimilarity.

a)

**Group active shallow**	**Av. Sim: 47.41**			
**Taxon**	**Av.Abund**	**Av.Sim**	**Contrib%**	**Cum.%**
Caprella spp.	3.83	12.55	26.48	26.48
Oligochaeta unid.	3.6	11.03	23.26	49.74
Phascolion (Phascolion) strombus	2.55	3.62	7.64	57.38
Lumbrineridae unid.	2.12	3.13	6.61	63.98
**Group inactive shallow**	**Av. Sim: 45.86**			
Syllidae unid.	4.16	6.24	13.61	13.61
Goodallia spp.	3.05	3.62	7.89	21.5
Lysidice unicornis	2.92	3.59	7.84	29.34
Lucinidae *spp.*	2.27	2.75	5.99	35.33
Phascolion (Phascolion) strombus	3.11	2.73	5.95	41.28
Bittium reticulatum	2.41	2.31	5.04	46.32
Branchiostoma lanceolatum	1.83	2.29	4.98	51.3
Gammaridea unid.	2.26	2.06	4.49	55.79
Nereididae unid.	2.22	2.06	4.48	60.27
**Group active deep**	**Av. Sim: 23.84**			
Gammaridea unid.	2.07	10.23	42.91	42.91
Eurycopidae unid.	1.19	3.52	14.76	57.67
Oligochaeta unid.	1.94	3.51	14.72	72.39
**Group inactive deep**	**Av. Sim: 39.89**			
Phascolion (Phascolion) strombus	2.95	4.26	10.67	10.67
Aponuphis bilineata	2.55	3.86	9.69	20.36
Gammaridea unid.	2.68	3.79	9.5	29.86
Oenonidae unid.	2.28	3.52	8.84	38.7
Glycera spp.	2.29	3.37	8.46	47.16
Lysidice unicornis	2.07	3.18	7.98	55.13
Echinoidea unid.	1.65	1.96	4.92	60.06

b)

**Shallow sites: active *vs.* inactive**	**Av. Diss = 77.11**				
	**Active**	**Inactive**			
**Taxon**	**Av.Abund**	**Av.Abund**	**Av.Diss**	**Contrib%**	**Cum.%**
Caprella sp.	3.83	0	4.48	5.81	5.81
Syllidae unid.	0.72	4.16	3.97	5.15	10.96
Oligochaeta unid.	3.6	0.78	3.46	4.49	15.45
Goodallia sp.	0	3.05	3.33	4.31	19.77
Lysidice unicornis	0	2.92	3.27	4.24	24
Phascolion (Phascolion) strombus	2.55	3.11	2.74	3.56	27.56
Bittium reticulatum	0	2.41	2.73	3.54	31.1
Lucinidae unid.	0	2.27	2.54	3.3	34.4
Lumbrineridae unid.	2.12	1.7	2.17	2.82	37.21
Polychaeta unid.	0	1.88	2.17	2.82	40.03
Anomura unid.	0	2.09	2.11	2.73	42.76
Nereididae unid.	0.73	2.22	1.98	2.57	45.33
Capitellidae unid.	0.93	2.06	1.97	2.55	47.89
Pisione sp.	1.99	1.5	1.96	2.54	50.43
Leptocheirus sp.	1.78	0.27	1.91	2.47	52.9
Branchiostoma lanceolatum	0.72	1.83	1.88	2.44	55.35
Leucothoe sp.	1.66	0.7	1.87	2.42	57.77
Hesionidae unid.	1.44	1.88	1.82	2.36	60.13
**Deep sites: active *vs.* inactive**	**Av. Diss = 86.41**				
	**Active**	**Inactive**			
Phascolion (Phascolion) strombus	0.36	2.95	4.45	5.15	5.15
Aponuphis bilineata	0	2.55	4.43	5.12	10.27
Oenonidae unid.	0	2.28	3.79	4.39	14.66
Lysidice unicornis	0	2.07	3.52	4.07	18.73
Glycera sp.	0.89	2.29	3.12	3.61	22.34
Oligochaeta unid.	1.94	0	3.08	3.57	25.91
Pereionotus testudo	0	1.74	2.85	3.3	29.21
Echinoidea unid.	0	1.65	2.84	3.29	32.5
Hyalinoecia tubicola	0.47	1.62	2.76	3.19	35.7
Gammaridea unid.	2.07	2.68	2.49	2.89	38.58
Bivalvia unid.	0	1.42	2.42	2.8	41.39
Scolecida unid.	0	1.46	2.42	2.8	44.19
Onuphis eremita	0	1.44	2.37	2.74	46.92
Syllidae unid.	0.36	1.36	2.27	2.63	49.55
Tanaidacea unid.	1.18	0.47	2.13	2.46	52.01
Eurycopidae unid.	1.19	0	2	2.32	54.33
Polyplacophora unid.	0	1.18	1.98	2.29	56.62
Anomura unid.	0	1.24	1.92	2.22	58.84
Cirratulidae unid.	0	1.08	1.86	2.16	60.99
